# Complex response inhibition and cognitive flexibility in school-aged Cypriot-Greek-speaking children who stutter

**DOI:** 10.3389/fpsyg.2022.991138

**Published:** 2022-11-18

**Authors:** Maria Paphiti, Eira Jansson-Verkasalo, Kurt Eggers

**Affiliations:** ^1^Department of Psychology and Speech-Language Pathology, University of Turku, Turku, Finland; ^2^Department of Rehabilitation Sciences, Speech-Language Pathology/Audiology Research Group, Ghent University, Ghent, Belgium; ^3^Department of Speech-Language Therapy and Audiology, Thomas More University College, Antwerp, Belgium

**Keywords:** inhibitory control, cognitive flexibility, executive function, stuttering, set-shifting, performance-cost

## Abstract

**Purpose:**

Over the last few years, research findings have suggested limitations in executive function (EF) of children who stutter (CWS) with the evidence being more consistent in studies with preschoolers (3–6  years old) than in studies with school-aged children (6–12  years old). The purpose of the current study was to assess complex response inhibition and cognitive flexibility in school-aged CWS and their non-stuttering peers.

**Methods:**

Participants, 19 CWS (mean age = 7.58  years, range 6.08–9.17) and 19 age-and gender-matched children who do not stutter (CWNS; mean age = 7.58  years, range 6.08–9.33), completed a visual task consisting of three task blocks. Analyses were based on response times and error percentages during the different task blocks.

**Results:**

All participants showed expected performance-costs in task block comparisons targeting complex response inhibition and cognitive flexibility. Significant group differences were found in measures of cognitive flexibility with CWS performing slower compared to CWNS (*p* = 0.02). Additionally, significant block × group interactions demonstrated that CWS, compared to CWNS, slowed down more (i.e., higher performance-cost) under both complex response inhibition (*p* = 0.049) and cognitive flexibility task conditions (*p* = 0.04 for no-set-shifting and *p* = 0.02 for set-shifting).

**Conclusion:**

These results are in line with some of the previous findings in school-aged CWS and suggest that CWS present lower performance in complex response inhibition and cognitive flexibility task conditions when compared to their non-stuttering peers.

## Introduction

Developmental stuttering is a complex neurodevelopmental and multifactorial disorder that usually first appears in children between the ages of 2.5 and 4 years with a prevalence rate of 1.4–1.44 and male-to-female ratio of 2.3:1, approaching 4:1 in adolescence (Craig et al., 2002). Its core characteristics are disfluencies that disrupt the natural-sounding flow of speech (i.e., single-syllable word and part-word repetitions, blocks, broken words and sound prolongations; Ambrose and Yairi, 1999). Stuttering is often accompanied by secondary physical behaviors and indications of a cognitive and emotional impact such as anxiety about speaking (Alm, 2004; American Psychiatric Association, 2013). Over the past decades, developmental stuttering has been linked to various domain-specific processes that are associated with motor, emotional, sensory, and speech-language development (e.g., Bernstein Ratner and Silverman, 2000; Silverman and Ratner, 2002; Dworzynski et al., 2007; Watkins et al., 2007; Ntourou et al., 2011; Jones et al., 2014). These findings led researchers to suggest the ‘multifactorial dynamic pathways theory’ as a possible explanation for the development of stuttering (Smith and Weber, 2017). Research has also demonstrated a strong relationship between language development and domain-general cognitive processes, i.e., executive functions (EFs; Blair, 2003; Conway and Pisoni, 2008; Müller et al., 2009).

EF is an umbrella term (Chan et al., 2008) encompassing top-down neurocognitive processes that are involved in the planning and execution of goal-driven actions (Miyake et al., 2000; Zelazo and Carlson, 2012; Diamond, 2013), especially in non-routine situations (Barkley, 2012, p. 7). Well-developed EFs provide children the ability to hold information for later use (working memory), avoid distractions (inhibitory control), shift and sustain attention to what is important (cognitive flexibility). They help people control their thoughts and actions in adaptive ways (self-regulation) to complete novel or complex tasks (Miller and Cohen, 2001), such as speech and language.

A child’s speech, language, and core EFs (i.e., working memory, inhibitory control, and cognitive flexibility) develop dramatically during the age of 2.5 to 4 years, which is roughly the same period during which developmental stuttering appears. These core EFs show different developmental trajectories (Best, 2010; Hughes, 2011) with working memory beginning to develop as early as 9 months of age (Diamond, 2013). Inhibitory control develops during the late preschool years (ages 3–5; Garon et al., 2008) along with some aspects of cognitive flexibility (Zelazo et al., 2003). All three EFs continue to develop during the elementary-school years and throughout adolescence (Cepeda et al., 2001; Best, 2010; Hughes, 2011; Carriedo et al., 2016).

Difficulties in the core EFs of working memory, inhibitory control, and cognitive flexibility (Pennington and Ozonoff, 1996; Miyake et al., 2000; Zelazo and Müller, 2011; Cragg and Chevalier, 2012; Diamond, 2013) have been linked to stuttering (Eggers et al., 2013; Anderson and Wagovich, 2017; Eggers and Jansson-Verkasalo, 2017; Eichorn et al., 2018). Despite the increasing number of researchers suggesting that weaker EFs are correlated with and may be a feature of stuttering (e.g., Choo et al., 2020), these cognitive factors are not yet clearly understood (Eichorn et al., 2019). Therefore, the purpose of the current study was to assess and compare children who stutter (CWS) to children who do not stutter (CWNS) in two core EFs: inhibitory control (specifically, complex response inhibition) and cognitive flexibility (specifically, the domain of set-shifting).

Inhibitory control is the ability to withhold or delay a response that is considered inappropriate under certain instructions or in novel or uncertain situations (Garon et al., 2008). Inhibitory control and working memory are related. High working memory load negatively impacts inhibitory control due to the limited resource of the first (Boucher et al., 2021). This may result in an increase of inappropriate behaviors (i.e., errors). There are three types of inhibitory control: (a) prepotent response inhibition, (b) resistance to distractor interference, and (c) resistance to proactive interference (Friedman and Miyake, 2004). Prepotent response inhibition refers to suppressing dominant or entrained responses. Inability to do so, is usually presented as premature responses or false alarms (e.g., Eggers et al., 2013). Resistance to distractor interference is explained as ignoring distractions surrounding a target stimulus (e.g., Eggers et al., 2012), while resistance to proactive interference refers to not allowing memories of previously learned rules to interfere with the execution of new rules (e.g., Postle et al., 2004). Prepotent response inhibition is considered complex if additional processing is required to both suppress a dominant response and execute a conflicting response (Anderson and Wagovich, 2017), i.e., not to withhold a motor response but instead to respond in a different manner that it is now considered appropriate.

Studies examining inhibitory control in relation to developmental stuttering are limited in number and have only examined prepotent response inhibition and complex prepotent response inhibition. Prepotent response inhibition in CWS has been investigated in six studies (Table 1) that used behavioral (Eggers et al., 2013, 2018; Piispala et al., 2016) and neurocognitive measures (Harrewijn et al., 2017; Piispala et al., 2017, 2018). The behavioral studies revealed no group differences between the CWS and CWNS (Piispala et al., 2016; Eggers et al., 2018), with the exception of Eggers et al. (2013) in which study children as young as 4 years old were included. In the two neurocognitive studies by Piispala et al., the researchers reported weaknesses in prepotent response inhibition, while in the third by Harrewijn et al., greater response inhibition skills for the CWS were reported. Overall, in two studies, no differences were reported between CWS and CWNS (Piispala et al., 2016; Eggers et al., 2018), in three studies, limitations were reported (Eggers et al., 2013; Piispala et al., 2017, 2018), and in one, a better performance was reported for CWS (Harrewijn et al., 2017).

**Table 1:** Summary of studies that used behavioral and neurocognitive measures to assess inhibitory control in CWS and CWNS.

**Study**	**n**	**Age**	**Task used**	**Type of inhibition**	**Domain and response**	**Type of measure**
Eggers et al., 2013	30 CWS 30 CWNS	4.83–10.0	Go/NoGo	Prepotent response	D: Visual R: Manual	Behavioral
Piispala et al., 2016	11 CWS 19 CWNS	5.67–9.5	Go/NoGo	Prepotent response	D: Visual R: Manual	Behavioral
Piispala et al., 2017	11 CWS 19 CWNS	5.67–9.5	Go/NoGo	Prepotent response	D: Visual R: Manual	Neurocognitive
Piispala et al., 2018	11 CWS 19 CWNS	5.67–9.5	Go/NoGo	Prepotent response	D: Visual R: Manual	Neurocognitive
Eggers et al., 2018	18 CWS 18 CWNS	7.33–10.91	Stop-signal	Prepotent response	D: Auditory R: Manual	Behavioral
Harrewijn et al., 2017	17 CWS 19 CWNS	9.0–14.0	Stop-signal	Prepotent response	D: Visual R: Manual	Neurocognitive
Anderson and Wagovich, 2017	41 CWS 41 CWNS	3.08–6.08	Grass-snow and Baa-meow	Complex response	D: Auditory R: Manual	Behavioral
Eggers and Jansson-Verkasalo, 2017	16 CWS 16 CWNS	6.33–9.83	Auditory Set-Shifting	Complex response	D: Auditory R: Manual	Behavioral

Complex response inhibition in CWS has been investigated in a behavioral study in younger (3.08–6.08 years; Anderson and Wagovich, 2017) and older (6.33–9.83 years) participants (Eggers and Jansson-Verkasalo, 2017). Both studies reported CWS to be less accurate, while only the Anderson and Wagovich study reported CWS to also be slower. Since both studies used auditory stimuli and several researchers have suggested auditory processing difficulties in CWS (Foundas et al., 2004; Hampton and Weber-Fox, 2008; Liotti et al., 2010; Jansson-Verkasalo et al., 2014), it is unclear whether the limitations in the performance of CWS were due to difficulties in auditory processing or complex response inhibition. Therefore, it is important to conduct further investigation to reach firm conclusions but with the use of tasks that measure complex response inhibition *via* the visual domain.

Cognitive flexibility is the ability to adapt to changing environments and flexibly shift between tasks or mental sets (Schuiringa et al., 2017). It involves top-down neurocognitive processes that enable us to sort information based on different dimensions such as color and form or to move from one rule to another (Garon et al., 2008; Nigg, 2017). Cognitive flexibility facilitates self-regulation (Nigg, 2017; Eichorn and Pirutinsky, 2021) and has been associated with both speech and language development (Deák, 2004; Crosbie et al., 2009). It is considered the “pinnacle of human cognition” (Logan, 2004), builds upon inhibitory control and working memory (Garon et al., 2008): in order to change a certain perspective, one needs to inhibit (deactivate) a previous perspective and load (activate) a new perspective into the working memory (Diamond, 2013). During speech/language planning and production, cognitive flexibility (along with the other two core EFs) allows us to transfer (shift) attentional focus from what is not important to what is important. One example during a conversation could be that it allows us to shift attention away from possible speech/language errors and quickly redirect attention in planning and producing new speech/language formulations.

Cognitive flexibility is frequently evaluated through task-switching and set-shifting tasks (Diamond, 2013), such as alternation-design or mixed-block design tasks (Gopher et al., 2000). Alternation-design tasks contrast the performance on blocks where one continually needs to alternate (switch) between tasks from trial to trial, with the performance on blocks in which one executes the same task on all trials. Mixed-block design tasks consist of repeated presentation of blocks in which the same task and shifts between tasks are mixed within one block. Both types of tasks attempt to reflect everyday situations such as when interacting in demanding communicative situations or when having to follow directions. In both cases, well-developed cognitive flexibility is crucial.

Thus far, only four behavioral studies have investigated cognitive flexibility in relation to developmental stuttering (Table 2). Two of the studies included preschool children (3.0–6.5 years old; Eichorn et al., 2018; Anderson et al., 2020). In these two studies alternation-design tasks were used and measured switching. The other two, included school-aged children (6.33–11.92 years old; Eggers and Jansson-Verkasalo, 2017; Eichorn and Pirutinsky, 2021). The Eggers and Jansson-Verkasalo study used a mixed-block-design task measuring set-shifting, while the Eichorn and Pirutinsky study, used an alternation-design task evaluating switching. Alterations were made in the task used in the Eichorn and Pirutinsky study in order to also measure set-shifting. Despite the differences in the study design (See Table 2), the results of all four studies showed limitations in cognitive flexibility for the group of CWS: preschool CWS were found to be slower, while older CWS were reported to be slower (Eichorn and Pirutinsky, 2021) or less accurate (Eggers and Jansson-Verkasalo, 2017).

**Table 2:** Summary of behavioral studies assessing cognitive flexibility in CWS and CWNS.

**Study**	**n**	**Age**	**Task used**	**Type of task**	**Domain and response**
Anderson et al., 2020	44 CWS 44 CWNS	3.0–5.92	Double Semantic and Perceptual Categorization	Alternation-design	D: Visual R: Manual
Eichorn et al., 2018	16 CWS 30 CWNS	3.0–6.5	Dimension Card Change Sort	Alternation-design	D: Visual R: Manual
Eggers and Jansson-Verkasalo, 2017	16 CWS 16 CWNS	6.33–9.83	Auditory Set-shifting	Mixed-block design	D: Auditory R: Manual
Eichorn and Pirutinsky, 2021	15 CWS 18 CWNS	8.0–11.92	Dimension Card Change Sort	Alternation-design with modifications	D: Visual R: Manual

In conclusion, findings on cognitive or attentional flexibility in CWS, even though limited, have suggested lower performance for CWS when compared to CWNS. These findings are of theoretical importance and can provide us with additional information about the multifactorial nature of stuttering and the variability within the stuttering population (Anderson et al., 2020).

Along with the findings in the area of EFs and developmental stuttering, a recently proposed model, the ‘executive function model of developmental stuttering’ (Anderson and Ofoe, 2019) tries to provide an explanation for the possible role of EFs in developmental stuttering. It states that the domain-specific processes, i.e., motor, sensory, emotional, and linguistic processes are dependent on and build onto domain-general EFs. Hence, any weakness in EFs, such as working memory, inhibitory control, or cognitive flexibility, might lead to problems in the domain-specific processes, such as speech. Taken all together, both evidence and the executive function theoretical model of developmental stuttering, seem to suggest a possible association between limitations in EFs and speech disfluencies.

The current study investigates complex response inhibition and cognitive flexibility in a combined manner with the use of a visual mixed-block design task. The choice for a visual task was to avoid the possibility of auditory processing difficulties in the CWS group affecting the results, as discussed in the study by Eggers and Jansson-Verkasalo (2017). Moreover, complex response inhibition tasks, in comparison to prepotent response inhibition tasks, are reported to be more challenging and sensitive to detecting group differences (Schuiringa et al., 2017), and more comparable to real-life activities (Aron, 2011; Diamond, 2013). Similarly, set-shifting tasks are challenging and require more complex skills because set-shifting “can be seen as an EF process operating on another EF process” (Garon et al., 2008).

The main research questions were:

1. Are CWS, as a group, slower and/or less accurate than CWNS in task-based assessments of complex response inhibition?
2. Are CWS, as a group, slower and/or less accurate than CWNS in task-based assessments of cognitive flexibility?

We hypothesized that CWS would be less efficient (i.e., higher response times and/or higher error percentages) under complex response inhibition and even more under cognitive flexibility task conditions, due to its increased complexity. Under complex response inhibition task conditions, responses need to correspond to a newly introduced rule. In the case of cognitive flexibility task conditions, responding becomes more complex since participants need to shift between responses based on two previously introduced rules. Lastly, we hypothesized that the performance of both groups would improve with age as suggested by the Amsterdam Neuropsychological Tasks (ANT; de Sonneville, 2009) literature (Huijbregts et al., 2002; Rommelse et al., 2007; Sterken et al., 2016).

## Materials and methods

### Participants

The final number of participants who met the inclusion criteria was 19 CWS (mean age 7.58 years; *SD* = 1 year; range = 6.08–9.17) and 19 CWNS (mean age 7.58 years; *SD* = 1.08 years; range = 6.08–9.33). The two groups were matched for gender (18 males and 1 female) and age (±3 months); the between-group age comparison was *t* (36) = 0.11, *p* = 0.91. Specific inclusion criteria for CWS were (a) a diagnosis of developmental stuttering by a speech-language pathologist, (b) a severity equivalent of at least “mild” on the Stuttering Severity Instrument-4 (SSI-4; Riley, 2009), and (c) no history of speech and/or language therapy other than for stuttering. The inclusion criteria for CWNS were (a) no parental concern regarding stuttering, (b) a severity equivalent of less than “mild” on the SSI-4, and (c) no history of speech and/or language therapy. Seven CWS (five males) and three CWNS (all males) were excluded from the study because they did not meet at least one of the inclusion criteria. Additional eligibility criteria for participating in the study (collected using parental questionnaires and interviews with the teachers) were the following: (a) monolingual Greek speakers with no other languages being spoken in their homes, (b) no known or questionnaire-reported psychological, neurological, or developmental problems, except for stuttering in the CWS group, and (c) normal hearing and normal or corrected-to-normal vision.

The parental educational level was determined based on the sum of the scores of the highest educational level of each of the two parents (*primary education* = 1, *high school* = 2, *1-or 2-year vocational college certificate* = 3, *university degree* = 4), as in a previous publication (Eggers et al., 2018). According to Rindermann and Baumeister (2015), parental educational behavior is more closely related to a child’s cognitive development than any other factor that determines socioeconomic status. A Mann–Whitney test (scores were not normally distributed) revealed no differences between the CWS (mean = 6.47, range = 3–8) and the CWNS (mean = 7.26, range = 4–8), *U* = 133.50, *p* = 0.17.

The data collection took place in Cyprus and was conducted by the first author, an ASHA certified speech-language pathologist, and EU-certified fluency specialist. Only children attending the mainstream school system with no teacher-or SLP-reported speech, language, and/or learning difficulties were included. None of the participants had received any speech-language therapy, other than for stuttering for the CWS. There are no standardized language tests for school-aged Greek-speaking children. Therefore, we administered the Bus Story Test, which is widely used in its unstandardized form in studies with Greek-speaking populations (e.g., Theodorou et al., 2016). We compared the two groups on 15 measures such as information [CWS: mean = 39, *SD* = 8.41 and CWNS: mean = 43, *SD* = 9.52, *t*(36) = −1.37, *p* = 0.18], subordinate clauses [CWS: mean = 9.05, *SD* = 4.02 and CWNS: mean = 8.53, *SD* = 3.96, *t*(36) = 0.41, *p* = 0.69], mean length of utterance (CWS: mean = 6.10, *SD* = 0.95 and CWNS: mean = 6.20, *SD* = 1.75, *U* = 163.50, *p* = 0.62), T-units (CWS: mean = 20.74, *SD* = 3.83 and CWNS: mean = 22.63, *SD* = 6.09, *U* = 151.00, *p* = 0.40), total number of words (CWS: mean = 125.05, *SD* = 27.02) and CWNS: mean = 133.11, *SD* = 23.79, *t*(36) = −1.09, *p* = 0.28, and no significant differences occurred between the two groups.

Stuttering severity was assessed through the SSI-4 for which we collected four speech samples from each participant on two different days to obtain a reliable representation of stuttering severity. Scores on the SSI-4 were calculated based on a total sample of minimum of 300 words. The following were considered “stuttering-like disfluencies”: (a) part-word repetitions (e.g., b-but), (b) single-syllable word repetitions (e.g., and and), (c) dysrhythmic phonation (specifically prolongation, e.g., “mmmmy” “cooookie”), blocks (e.g., “#table”), and broken words (e.g., “o#pen”; Ambrose and Yairi, 1999). Seven CWS were classified as *mild*, nine as *moderate*, two as *severe*, and one as *very severe*. CWS had an average of 6.36 (*SD* = 3.00) stuttering-like disfluencies per 100 syllables, while CWNS had an average of 0.83 (*SD* = 0.42).

Participants’ hearing was screened using bilateral screening tone-audiometry at 500, 1000, 2000, and 4,000 Hz (Amplivox 240, United Kingdom) with signals presented at 20 dB. All participants passed the hearing screening. Hand preference was determined using parental questionnaires. Two CWS and two CWNS were reported to be left-handed, while all other participants were right-handed.

To preclude cognitive group differences, the vocabulary and block design subtests of the Wechsler Intelligence Scale for Children-Third Edition (Wechsler, 1997) were administered. This test consists of seven performance and six verbal subtests. Vocabulary (verbal) and block design (performance) subtests were chosen because previous studies have shown that the scores of these two subtests correlate well with the overall score of the test (Groth-Marnat, 1997). For the vocabulary subtest, participants are required to explain the meaning of single words, while in the block-design subtest, participants are required to rebuild, as quickly as possible, a geometrical pattern with the use of four to nine two-colored cubes. The mean vocabulary standard scores were 11.80 for the CWS and 14 for the CWNS. The mean block-design standard scores were 12 for the CWS and 12.89 for the CWNS. No significant group differences were found in the vocabulary subtest (*U* = 114.50, *p* = 0.053) nor in the block design subtest (*U* = 148, *p = 0*.35), although four CWS scored below the standard score 10 (5,8,8,6) in the WISC-3 test (Vocabulary subtest) that was also administered.

### Materials

Two tasks from the ANT (de Sonneville, 2009) were used: the baseline speed task and Response Organization Objects (ROO). The ANT is a neuropsychological battery that consists of 38 tasks. It is designed to evaluate different EF processes by measuring the participants’ speed and accuracy of manual responses in different tasks. Over the last years, researchers have used ANT tasks to evaluate EF processes related to developmental stuttering (Eggers et al., 2013; Piispala et al., 2016; Eggers and Jansson-Verkasalo, 2017) as well as in relation to other disorders (Stoit et al., 2013; Sterken et al., 2016; Schuiringa et al., 2017). Several studies have shown that the ANT has good validity (de Sonneville, 1999, 2005; Polderman et al., 2007; Oerlemans et al., 2016) and test–retest reliability (Huijbregts et al., 2002; Oerlemans et al., 2016).

#### Baseline speed task

Prior to undertaking the ROO task, participants were administered the baseline speed task (de Sonneville, 2009), a simple computer-based response-time task. The purpose of administering the baseline speed task first was twofold: (a) for participants to be familiarized with computerized testing and (b) to eliminate the possibility of any response time differences that would have confounded the results of the ROO task. Per the instruction manual, during the first part, participants were asked to place the index finger of their non-dominant hand on the corresponding response key. They were instructed to press the response key as quickly and accurately as possible when the centralized white fixation cross on the black screen changed into a centralized white square. The second part of the task was similar and completed with the index finger of their dominant hand. An instruction session of two trials was followed by a practice session of 10 trials. The experimental session consisted of 32 trials for both the right and left index fingers. The signal duration was variable until a response was provided. For a valid response, response times had to fall between 150 ms and 4,000 ms after stimulus onset. Post-response intervals varied randomly from 500 ms to 2,500 ms.

#### Experimental task: Response Organization Objects

The ROO task is a mixed-block design task and is used to assess complex response inhibition and cognitive flexibility in a combined manner. It was designed for 4-to 12-year-old children and is the visual variant of the auditory set-shifting task (de Sonneville, 2009) used in the Eggers and Jansson-Verkasalo study. It consists of three different blocks, and each participant needs to complete all three. Participants were asked to place both index fingers on the two response keys. Each block had a different stimulus–response mapping, meaning that participants in each block were instructed to respond differently when the stimulus appeared on the screen.

**Figure 1:**
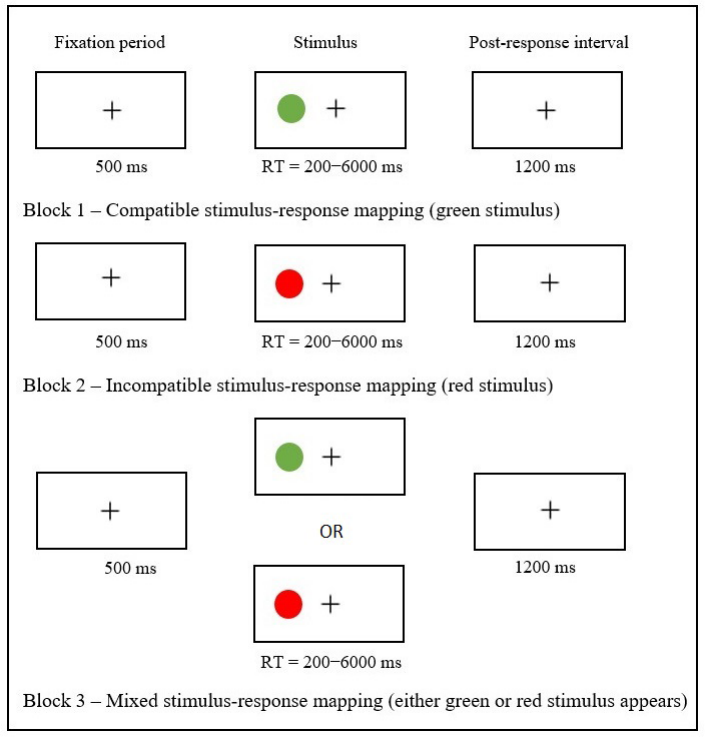
Schematic overview of the Response Organization Objects task.

Block 1 (hereafter compatible block) used compatible stimulus–response mapping. The stimulus was a green ball that appeared either on the left or on the right side of the screen. Participants were expected to press the corresponding response key, either left or right, when the stimulus appeared. The compatible block provided a baseline measure of speed and accuracy; Block 2 (hereafter incompatible block) used incompatible stimulus–response mapping. The stimulus was a red ball. Participants were asked to press the opposite response key when a red ball appeared. These two blocks did not require any set-shifting to complete them. Block 3 (hereafter mixed block), on the other hand, had mixed stimulus–response mapping. Either a green or a red ball appeared on the left or the right side of the black screen, and participants were instructed to press the corresponding response key if a green ball appeared or the opposite response key if a red ball appeared (Figure 1). In this block, both sets of rules introduced in the two previous blocks needed to be held in working memory, and participants were required to shift their attention from one to the other. In all three blocks, signals were presented until a response was given between 200 ms and 6,000 ms in a fixed randomized order. If a response was not given within this timeframe, it was counted as an omission and the signal was replaced by a new trial.

A fixation period of 500 ms was used before every stimulus presentation. The post-response intervals were fixed at 1200 ms. The compatible and incompatible blocks consisted of an instruction session (two trials), followed by a practice session (eight trials), and an experimental session (30 trials each). The mixed block began with an instruction session (two trials), followed by a practice session (16 trials) and an experimental session (60 trials). In the experimental session, 30 trials were compatible and 30 were incompatible and there were 16 shifts from compatible to incompatible and 15 shifts from incompatible to compatible stimulus. Prior to each experimental block and throughout the three instruction and practice sessions, all participants were instructed to press the response key as quickly and as accurately as possible. The experimental sessions began only when the task had been explained and the participants indicated that everything was clear.

As per the task manual, (a) complex response inhibition was evaluated by comparing response times (speed) and error percentages (accuracy) between the compatible and the incompatible block; (b) cognitive flexibility was evaluated by comparing response times (speed) and error percentages (accuracy) between the compatible and the mixed block (compatible part); expanding on the manual’s recommendations, we distinguished between the set-shifting and the no-set-shifting compatible trials of the mixed block as set-shifting may impose different demands on cognitive flexibility compared to no-set-shifting. Even though this was not a recommendation of the manual, we further evaluated cognitive flexibility by comparing response times (speed) and error percentages (accuracy) between the trials from the incompatible block and the incompatible part of the mixed block (both set-shifting and no-set-shifting trials) to limit the likelihood of inhibitory control (which has been documented previously to be lower in CWS) impacting the results.

### Procedure

All participants were volunteers. They were recruited through an open call to participate in the study, sent to all private and public schools under the jurisdiction of the Cyprus Ministry of Education, Culture, Sports, and Youth; the open call was also provided to all registered speech-language pathologists on the island. The participants were tested in rooms where sounds and other distractions were minimal. Stimuli were presented on an LG laptop R510 with a 15.4. screen, and the laptop was placed on a table in front of a plain wall, approximately 18 in from the participant. Participants were seated facing the screen. Data collection required two sessions of 35to45minutes each, and tests were always administered in the same order. All procedures were approved by the Cyprus National Bioethics Committee and the Center of Educational Research and Evaluation of the Cyprus Pedagogical Institute. Participation consent forms were collected for all participants.

### Data analyses

Prior to testing the hypotheses, a Mann–Whitney test was used to determine whether there were any between-group response time differences in the baseline speed task that would have confounded the results of the ROO task. This test was selected over a *t*-test because the response times of the two groups were not normally distributed.

To compare the efficiency of complex response inhibition between the groups, two pairs of mixed analyses of covariance (ANCOVA) were used, with response times and error percentages as their dependent variable. In both analyses, group (CWS vs. CWNS) was set as the between-subjects variable, block (compatible vs. incompatible) as the within-subject repeated-measures variable, and age as a covariate.

To compare the efficiency of cognitive flexibility between the two groups, two pairs of mixed ANCOVAs with the same dependent variables—response time and error percentage—were conducted. In all the analyses, group was set as the between-subjects independent variable and age as a covariate. Block was set as the within-subjects repeated-measures variable with three levels. For the comparisons between the compatible and mixed block (compatible part), the levels were (1) compatible block, (2) compatible trials of the mixed block without set-shifting, and (3) compatible trials of the mixed block with set-shifting. For the comparisons between the incompatible and mixed block (incompatible part), the levels were (1) incompatible block, (2) incompatible trials of the mixed block without set-shifting, and (3) incompatible trials of the mixed block with set-shifting. The analyses were repeated with the Vocabulary subscores of the WISC-3 as a covariate, but because results were comparable with the results of the analyses with age as a covariate, they are not reported.

For each of the above analyses, the normality assumption was checked by subjecting the residuals to a Shapiro–Wilk test; if the residual distribution for any dependent variable was not normal, appropriate data transformation was performed based on the observed shape of the variable’s distribution. For all analyses, the residuals for error percentage deviated from normality; the residuals for response time were normally distributed except for the residuals of the incompatible block. Skewed data were transformed by a power transformation. After transformation, all residuals followed a normal distribution for all response times (except for the incompatible block in the analysis comparing the compatible with the incompatible block). For the error percentages, residuals did not follow a normal distribution. Therefore, the results for the latter should be treated with caution, although ANOVA is known to be robust to violations of normality (Field, 2009).

To detect possible outliers, the distributions of the dependent variables (response times and error percentages) were checked with the criterion of ±3 standard deviations from the mean (Howell, 1998). Outlier detection was also performed on mean response times (*cf.* Eggers and Jansson-Verkasalo, 2017). No outliers were detected in any of the analyses.

The effects of the age covariate were investigated with correlation tests between age and change in response times (hereafter performance-cost) from the compatible block to the incompatible or the mixed block, or from the incompatible block to the mixed block. The correlations were performed using Spearman’s correlation analysis when the distribution of the variables deviated from normality or Pearson’s correlation analysis when the variables followed a normal distribution.

Based on our hypotheses, the main effects and interactions in all ANCOVAs were planned. Correlations with age were explored by two-tailed *post hoc* tests. Additionally, the relation between stuttering severity and performance-cost was investigated with Kendall rank correlation analysis. In these cases, Bonferroni correction was applied for multiple comparisons. The significance level for all analyses was *α* = 0.05. Data analysis was conducted using SPSS (Statistical Package for the Social Sciences – Version 25 for Windows, IBM, Corp., Armonk, NY, United States).

## Results

The mean response times of the baseline speed task showed no significant group differences between CWS (*M* = 479 ms; *SD =* 152) and CWNS (*M* = 435 ms; *SD =* 58), *U* = 171.00, (*p* = 0.80).

Table 3 presents the means and standard deviations for response times (in ms), and error percentages for each combination of the group and block factors for Blocks 1 (compatible), 2 (incompatible), and 3 (compatible and incompatible part with and without set-shifting) of the ROO task.

**Table 3:** Means and standard deviations for response times (in ms) and error percentages for each combination of the group and block factors for Block 1, Block 2, and Block 3 (compatible and incompatible parts with and without set-shifting) and for the two groups of participants.

	**Measure**	**Response times**		**Error percentages**	
	**Group**	**CWS**	**CWNS**	**CWS**	**CWNS**
**Block 1 (30 compatible trials)**	**Mean**	538	530	2.81%	1.23%
	**SD**	156	80	3.38%	1.99%
**Block 2 (30 incompatible trials)**	**Mean**	846	713	7.37%	5.26%
	**SD**	369	147	5.62%	5.91%
**Block 3 (compatible) (14 no-set-shifting trials)**	**Mean**	1,090	891	5.64%	3.38%
	**SD**	461	153	9.98%	5.52%
**Block 3 (compatible) (15 set-shifting trials)**	**Mean**	1,254	1,005	9.12%	12.63%
	**SD**	521	207	9.99%	11.09%
**Block 3 (incompatible) (14 no-set-shifting trials)**	**Mean**	1,175	889	3.76%	8.37%
	**SD**	460	163	2.66%	4.30%
**Block 3 (incompatible) (16 set-shifting trials)**	**Mean**	1,247	981	10.20%	7.49%
	**SD**	508	203	11.07%	7.20%

### Complex response inhibition

**Figure 2:**
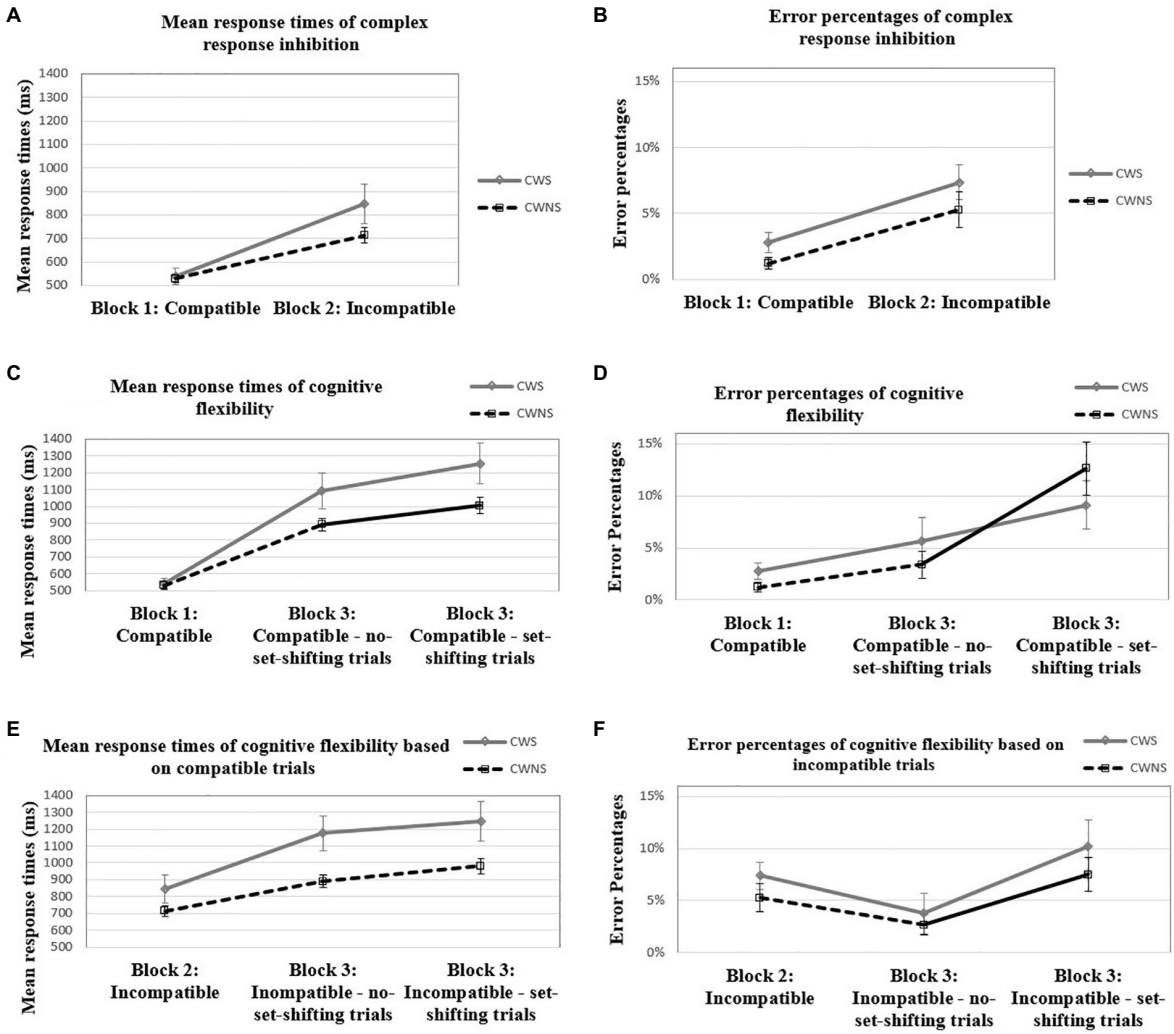
Mean response times (in ms) and error percentages of complex response inhibition **(A, B)**, mean response times (in ms) and error percentages of cognitive flexibility based on compatible trials **(C, D)**, and mean response times (in ms) and error percentages of cognitive flexibility based on incompatible trials **(E, F)** for the CWS and CWNS groups.

For the comparison of response times between the compatible and incompatible block, the between-subjects factor of group was not significant, *F*(1, 35) = 0.34, *p* = 0.57, partial *η*^2^ = 0.01. However, the block × group interaction was significant, *F*(1, 35) = 4.53, *p* = 0.04, partial *η*^2^ = 0.11. Further investigation revealed that, while both groups were slower in the incompatible than in the compatible block, CWS slowed down significantly more than CWNS, *t*(24.09) = −2.08, *p* = 0.049, Cohen’s *d* = −0.67, something that indicates higher performance-cost for the CWS (see Figure 2A). The effect of age was also significant, *F*(1, 35) = 10.75, *p* < 0.001, partial *η*^2^ = 0.23. A two-tailed Spearman’s correlation analysis indicated that the change in response times from the compatible to the incompatible block was negatively correlated with age for the CWS group, *ρ* = −0.49, *p* < 0.005, but not for the CWNS group, *ρ* = −0.24, *p* = 0.30. Nevertheless, there was a substantial overlap in the 95% confidence intervals between CWS [−0.772, −0.046] and CWNS [−0.626, 0.240].

For the comparison of error percentages between the compatible and the incompatible block, the between-subjects factor of group was not significant, *F*(1, 35) = 2.50, *p* = 0.12, partial *η*^2^ = 0.07. The block × group interaction was not significant, *F*(1, 35) < 0.001, *p* = 1.00, partial *η*^2^ < 0.001 (Figure 2B). The effect of age was not significant, *F*(1, 35) = 0.32, *p* = 0.58, partial *η*^2^ = 0.01.

### Cognitive flexibility

For the comparison of response times between the compatible and the mixed block (compatible part), the between-subjects effect for the group factor was not significant, *F*(1, 35) = 0.60, *p* = 0.44, partial *η*^2^ = 0.02. However, the block × group interaction was significant, *F*(1.54, 54.02) = 5.16, *p = 0*.01, partial *η*^2^ = 0.13. Further investigation revealed that, while both groups were slower in the mixed block (compatible part) than in the compatible block, CWS slowed down significantly more than CWNS, something that indicates higher performance-cost for the CWS (Figure 2C). Higher performance-cost for the CWS compared to the CWNS was present both when there was no set-shifting (i.e., a compatible trial preceded a compatible trial), *t*(21.71) = −2.23, *p* = 0.04, Cohen’s *d* = −0.08, and when there was set-shifting (i.e., an incompatible trial preceded a compatible trial), *t*(23.71) = −2.44, *p* = 0.02, Cohen’s *d* = 0.47. The effect of age was significant *F*(1, 35) = 13.19, *p < 0*.001, partial *η*^2^ = 0.27. Two-tailed Pearson’s correlation analyses indicated a significant negative correlation between age and performance-cost from the compatible to the mixed block (compatible part) for the CWS group, *r* = −0.676, *p* < 0.005, when there was no set-shifting, and *r* = −0.623, *p* = 0.01, when there was set-shifting. For the CWNS group, *r* = −0.232, *p* = 0.68, when there was no set-shifting, and *ρ* = −0.077, *p* = 1.00, when there was set-shifting. The 95% confidence intervals of the two groups showed substantial overlap both for the no set-shifting, *ρ* *=* −.077 (CWS: [−0.87, −0.32]; CWNS: [−0.621, −0.25]) and the set-shifting trials (CWS: [−0.84, −0.24]; CWNS: [−0.51, −0.39]).

For the comparison of response times between the incompatible and the mixed block (incompatible part), the between-subjects effect for the group factor was significant, *F*(1, 35) = 6.23, *p* = *0*.02, partial *η*^2^ = 0.15, with the CWS being slower than CWNS. The block × group interaction was significant, *F*(2, 70) = 3.69, *p* = *0*.03, partial *η*^2^ = 0.10. This interaction was investigated with a simple main effects analysis that indicated that CWS were slower than CWNS in the mixed block (compatible part) for both the no set-shifting (*p* = *0*.01) and the set-shifting trials (*p* = *0*.02); in the incompatible block, the two groups did not differ in speed, *p* = 0.14 (Figure 2E). In addition, while both groups were slower in the mixed block (incompatible part) than in the incompatible block, CWS slowed down significantly more than CWNS (i.e., there was higher performance-cost for the CWS group) when there was no set-shifting *t*(24.18) = −2.18, *p* = 0.04, Cohen’s *d* = −0.71; the two groups had comparable performance-cost when there was set-shifting, *t*(25.87) = −1.96, *p* = 0.06, Cohen’s *d* = −0.64. The effect of age was significant, *F*(1, 35) = 14.16, *p* < *0*.001, partial *η*^2^ = 0.29. Two-tailed Pearson’s correlation analyses indicated significant negative correlation between age and performance-cost from the incompatible to the mixed block (incompatible part) only for the CWS group when there was set-shifting, *r* = −0.549, *p* = 0.03, but not when there was no set-shifting, *r* = −0.418, *p* = 0.15. The 95% confidence intervals of the two groups showed substantial overlap both for the no set-shifting (CWS: [−0.73, −0.05]; CWNS: [−0.58, 0.30]), and the set-shifting trials (CWS: [−0.80, −0.13]; CWNS: [−0.76, 0.03]).

For the comparison of error percentages between the compatible and the mixed block (compatible part), no significant differences between the groups were observed, *F*(1, 35) = 0.31, *p* = 0.58, partial *η*^2^ = 0.01. The interaction between block and group was not significant, *F*(2, 70) = 1.73, *p* = 0.19, partial *η*^2^ = 0.05 (Figure 2D). The effect of age was not significant, *F* = (1, 35) = 0.41, *p* = 0.52, partial *η*^2^ = 0.01. The two groups had comparable performance-cost both when an incompatible trial preceded a compatible trial, *t*(36) = 1.46, *p* = 0.15, and when a compatible trial preceded the compatible trial, *U* = 163.50, *p* = 0.61.

For the comparison of error percentages between the incompatible and the mixed block (incompatible part), no significant differences between the groups were observed, *F*(1, 35) = 1.42, *p* = 0.24, partial *η*^2^ = 0.04. The interaction between block and group was not significant, *F*(2, 70) = 0.17, *p* = 0.85, partial *η*^2^ < 0.005 (Figure 2F). The effect of age was not significant, *F* = (1, 35) = 0.10, *p* = 0.76, partial *η*^2^ < 0.005. The two groups had comparable performance-cost both when a compatible trial preceded the incompatible trials, *t*(36) = −0.17, *p* = 0.86, and when an incompatible trial preceded the incompatible trials, *U* = 157.50, *p* = 0.50.

### Investigation of the correlation between stuttering severity and performance-cost

The relationship between severity of stuttering and the performance cost in response times for the analyses of complex response inhibition, and of cognitive flexibility was investigated with Kendall rank correlation analyses, which revealed non-significant results (*p* values between 0.24 and 1.00).

## Discussion

The current study evaluated whether 6-to 9-year-old CWS show limitations in inhibitory control and cognitive flexibility when compared to age-and gender-matched CWNS, as suggested by a growing body of research (e.g., Eggers and Jansson-Verkasalo, 2017; Piispala et al., 2017, 2018; Eichorn and Pirutinsky, 2021). Our key findings were that CWS, as a group and in comparison to CWNS, (a) had a higher performance-cost (i.e., slowed down more) in all measures of complex response inhibition and cognitive flexibility (significant block x group interactions), and (b) were significantly slower in the additional measures of cognitive flexibility (group differences) in which we attempted to control for the effects of inhibitory control.

### Complex response inhibition is slower in CWS

Our first hypothesis was that CWS would be less efficient than CWNS in task conditions that require complex response inhibition as documented by higher response times and higher error percentages. Indeed, there were significant differences between the two groups as a function of task (significant block × group interaction), with CWS slowing down more than CWNS in the incompatible block (complex response inhibition). Contrary to our hypothesis, the two groups did not differ in terms of accuracy. Eggers and Jansson-Verkasalo (2017) using the auditory set-shifting task, with participants of similar age range (6.33–9.83 years old), also found a block × group interaction for complex response inhibition for error percentages but not for speed. The difference between the results may partly be due to the modality used for the stimulus presentation—auditory for the auditory set-shifting task by Eggers and Jansson-Verkasalo (2017) versus visual for the ROO task. Namely, many studies even though not all (Kaganovich et al., 2010), have reported differences in auditory processing between individuals who stutter and who do not stutter (Foundas et al., 2004; Hampton and Weber-Fox, 2008; Liotti et al., 2010; Jansson-Verkasalo et al., 2014; Ismail et al., 2017). The auditory and visual systems differ in terms of how acoustic and visual information are being perceived and processed (e.g., Gomes et al., 2007; Lin et al., 2017). The neural networks engaged in performing auditory and visual tasks are reported to be similar (Bell et al., 2020), but the exact brain regions involved (within the networks) and their contribution may vary according to the stimulus modality (Lee et al., 2014). Furthermore, task-specific differences, such as stimulus presentation time and response type may have also contributed to the different findings between the two tasks. In the current ROO task, the on-screen stimulus presentation time varied between 200 and 6,000 ms, with the stimulus disappearing once a response was given. In the auditory set-shifting task, the stimulus presentation time was shorter, only 100 ms. Also, the rule that had to be applied in the incompatible block of the auditory set-shifting task (press twice after a single tone and once after a double tone) was more demanding compared to that of ROO (press the opposite response key). It could be that CWS, due to these increased demands of the auditory task, were not able to slow down sufficiently to maintain similar accuracy levels as CWNS.

Our hypotheses get support from the study by Anderson and Wagovich (2017), who used two alternation-design tasks with auditory stimuli. They reported CWS exhibiting longer response times as was the case in our study. In contrast to our study, they also reported a lower accuracy for the CWS in the task that targeted complex verbal response inhibition with the use of nonverbal stimuli (animal sounds). The difference between our findings may be due to the domain of stimulus-presentation but also to the task design differences. Additionally, the participants in the Anderson and Wagovich study were preschoolers (3.08–6.08 years old), a period of great development in the EFs but also a period during which CWS were reported to present with impaired sensorimotor learning compared to CWNS (Kim et al., 2020). Finally, our results are corroborating parental questionnaire-based findings (Eggers et al., 2010; Ntourou et al., 2018) as well as Go/NoGo-findings (Piispala et al., 2017, 2018) suggesting weaknesses in inhibitory control in CWS.

In sum, our results showed that CWS were less efficient than CWNS in task conditions that require complex response inhibition as documented by higher response times. We acknowledge that limitations in working memory may have contributed to the slowing down of CWS. Although it may seem more logical to investigate EFs *via* the auditory domain as it relates more directly to speech and language rather than *via* the visual domain, the possibility of auditory processing difficulties may interfere with the results, which is not the case when EFs are investigated *via* the visual domain.

### Cognitive flexibility is slower in CWS

Our second hypothesis was that CWS, compared to CWNS, would be slower and less accurate under cognitive flexibility task conditions. We have evaluated cognitive flexibility by conducting two sets of analyses: (a) compatible block vs. mixed block (compatible trials), and (b) incompatible block vs. mixed block (incompatible trials). In the first set of analyses, comparing the compatible trials, a significant block × group interaction was observed for response times (an index of speed), but not for error percentages. In the second set of analyses, comparing the incompatible trials, as hypothesized, differences were more evident than in the first set of analyses (significant group differences), with CWS being slower than CWNS.

Our findings are partially in agreement with Eggers and Jansson-Verkasalo (2017) who reported limitations for CWS in terms of accuracy but not in terms of speed. In both studies, a mixed-block design task was used, and therefore, comparisons can be made more safely. Our results are consistent with the findings of Eichorn et al. (2018), Anderson et al. (2020), and Eichorn and Pirutinsky (2021), who all reported longer response times for CWS. In the first two studies, alternation-design tasks were used with preschool age children and despite the difference in the age of the participants and the task design, similar results were reported as in the current study. In the third study (Eichorn and Pirutinsky, 2021), a modified version of the Dimension Change Card Sort Task (an alternation design task) was used that included a mixed block. Participants were school-aged children. The authors reported slower performance for the CWS in the mixed block. In both studies (the current and the Eichorn and Pirutinsky’s), the increased slowing down of the CWS compared to the CWNS was observed in the mixed block indicative of a higher performance-cost for the experimental group. A possible explanation for this could be that in mixed blocks, the alternation between set-shifting and no set-shifting is random. Under these conditions, participants experience high levels of uncertainty (Los, 1996) because they are in the position of not knowing if set-shifting or no-set-shifting is required. Due to this uncertainty, additional processing time is required for task-set-reconfiguration (Rogers and Monsell, 1995; Sohn and Carlson, 2000). Task-set-reconfiguration is the loading of the new rule into the working memory and the inhibition of processing of the old rule that no longer applies (Schmitter-Edgecombe and Langill, 2006). Working memory enables the maintenance of the two mental sets in an active state and inhibition enables the shifting between the two mental sets. Together, they are the main contributors to cognitive flexibility (Diamond, 2006).

### Older children have lower performance-costs (response time)

Our third hypothesis was that the performance of both participant groups would improve with age as a result of EF maturation, as suggested by the ANT literature (Huijbregts et al., 2002; Rommelse et al., 2007; Sterken et al., 2016). This hypothesis was partially corroborated by significant negative correlations between age and performance-costs for the CWS but not for the CWNS for both complex response inhibition (compatible block vs. incompatible block) and cognitive flexibility (compatible block vs. mixed block [compatible part] and incompatible block vs. mixed block [incompatible part]). Despite the significant correlations for the CWS and the non-significant ones for the CWNS, in all cases there was a large overlap of the confidence intervals, something that suggests that the two groups did not behave differently; arguably, with a bigger sample size a negative correlation between age and response times would have reached significance for the CWNS as well. In a previous study, that of Rocha et al. (2019), the EFs of CWS and CWNS (7–12 years old) were evaluated using a paper and pencil performance test. The researchers reported that the younger subgroup of CWS (7–9 years old) required longer response times and made more errors compared to their age-and gender-matched CWNS, while this was not the case when comparisons were made between the two older subgroups (10–12 years old). This finding allowed them to claim a possible slower maturation of EFs in CWS. Other researchers have made similar claims specifically in regard to inhibitory control, that is, CWS may improve and eventually “catch up” in performance the CWNS (Anderson and Ofoe, 2019, p. 309), something that might also be the case for cognitive flexibility. This could explain why studies with older participants (7–11 years old) reported either a comparable performance between CWS and CWNS for inhibitory control (Eggers et al., 2018) or a better performance for the CWS (9–14 years old; Harrewijn et al., 2017). A longitudinal study could elucidate whether the developmental trajectory of these two EFs differs between CWS and CWNS or not.

We also reported a non-significant relationship between stuttering severity and performance-cost although one might speculate that the higher the stuttering severity, the higher the performance-cost would be in both measures of the EF. Kraft et al. (2014) documented a similar type of relationship between parental ratings of executive control and the extreme ends of the stuttering severity range, i.e., very mild versus very severe, but this was less clear for moderate stuttering severity levels. The lack of these extreme stuttering severity levels in our sample (no child with a very mild and only one child with a severe and one with a very severe stuttering severity score) as well as a different way of measuring stuttering severity (parental rating versus SSI-4 score in our sample) might explain this lack of correlation.

### Theoretical implications

The findings of the current study suggest less efficient (slower) complex response inhibition and cognitive flexibility in CWS. This finding aligns with an increasing number of findings suggesting attentional regulation difficulties (Karrass et al., 2006; Jones et al., 2014) and attentional weaknesses in CWS (Eggers and Jansson-Verkasalo, 2017; Eichorn et al., 2018; Anderson et al., 2020). Both inhibitory control (e.g., Engelhardt et al., 2010, 2013) and cognitive flexibility (e.g., Deák, 2004; Crosbie et al., 2009) have been linked to speech-language planning and execution. They facilitate error detection prior to articulation and flexibly shifting one’s attention away from errors during articulation, resulting in increased fluency; attentional weaknesses on the other hand, may result in poor error monitoring, leading to disfluencies (Choo et al., 2020).

The fact that CWS slowed down significantly in both the complex response inhibition and in the cognitive flexibility measures, may reflect CWS needing to reduce their speed more to maintain similar levels of accuracy with CWNS. In other words, they might need additional time to regulate their behavior during attention-demanding situations, possibly also to plan and execute fluent speech and language (Anderson and Ofoe, 2019).

**Figure 3:**
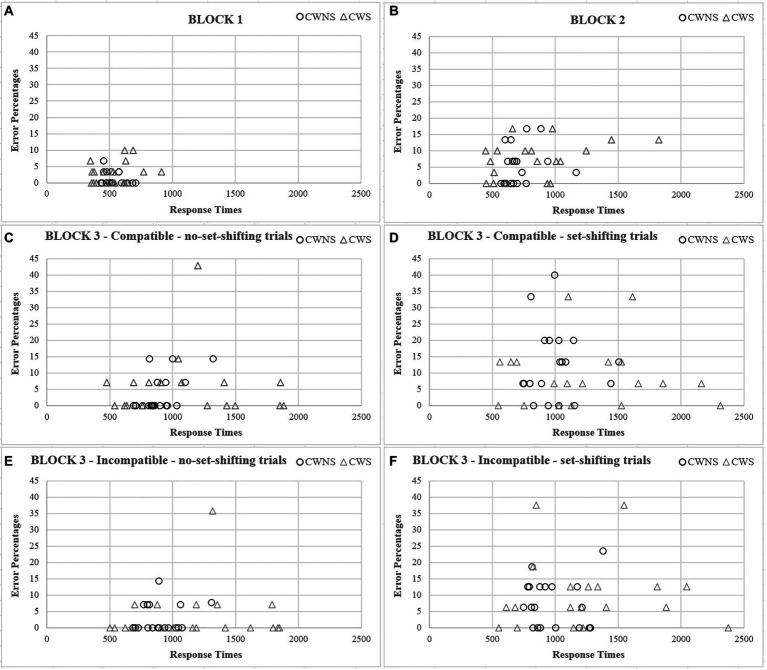
Scatterplots between speed and accuracy as a function of group for each block **(A, B)** and breakdowns of the mixed block **(C–F)**.

Our results also seem to support the claim of greater heterogeneity in the general stuttering population (e.g., Anderson and Ofoe, 2019; Singer et al., 2020). Despite our strict inclusion criteria, there was greater variability in both speed and accuracy in all blocks and breakdowns of the mixed block for the CWS group (Figure 3).

Although we elaborated on the possibility of less efficient EFs contributing to stuttering, our used methodology was not aimed at causality. Alternative explanations could be that both stuttering and impaired EFs share a common underlying neurological mechanism or that our findings are influenced by previously reported limitations in complex motor skills and sensorimotor learning in people who stutter (e.g., Loucks and De Nil, 2006; Smits-Bandstra et al., 2006; Kim et al., 2020). It is also possible, that CWS slowed down more than CWNS, because they needed additional time for adapting their response style to the needs of the task (see also Eggers et al., 2013) or because they had greater concern about errors (see also Eichorn et al., 2018).

Finally, our results on inhibitory control provide additional insights that may fill the gap between existing behavioral and neurocognitive studies. The results from the existing behavioral studies targeting prepotent response inhibition (Eggers et al., 2013; Piispala et al., 2016; Eggers et al., 2018; except for the Eggers et al., 2013 study in which children as young as 4 years old were included), reported comparable performance for the stuttering and the non-stuttering children, something that does not agree with the results from the neurocognitive studies (Piispala et al., 2017, 2018). In addition, the two behavioral studies that evaluated complex response inhibition, even though they reported limitations in EFs for the CWS group, similarly to the neurocognitive studies, they used auditory tasks with the likelihood of auditory processing difficulties impacting the results (Anderson and Wagovich, 2017; Eggers and Jansson-Verkasalo, 2017). Lastly, our results on cognitive flexibility are also of great importance, given the limited number of existing studies examining this EF in the stuttering population.

### Limitations

The lack of standardized language testing for Greek-speaking Cypriot children or a formal assessment of ADHD might be considered a caveat. However, for none of the participants there was any parental concern about language, learning or any other developmental problems. In addition, all the children’s teachers, and speech-language pathologists (for the CWS) were interviewed to rule out any co-occurring disorders, such as ADHD and, comparison of both participant groups on the different measures of the Bus Story Test did not yield any group-differences.

The male-to-female ratio (18, 1) does not adequately reflect the proportion typical in stuttering (3, 1 to 5, 1). Therefore, one could argue that the current findings are more a reflection of performance in school-age boys who stutter rather than children who stutter in general.

Finally, the additional measure of cognitive flexibility (a comparison of the incompatible trials of Block 3 with the trials of Block 2) goes beyond the suggested standard measure of cognitive flexibility and would benefit from further validation in the future.

## Conclusion

In sum, the results of the current study show a higher performance-cost (increased slowing down) for the CWS group under both complex response inhibition and cognitive flexibility task conditions. As hypothesized, differences were more evident (significant group differences) in measures of cognitive flexibility than in measures of complex response inhibition. These findings partly corroborate earlier research findings reporting slower performance for CWS in tasks targeting complex response inhibition and cognitive flexibility and suggest a possible relation between EFs and developmental stuttering.

Future EF studies would ideally include more participant within a broader age range, allowing for a better insight in how the developmental pathway in CWS compares to CWNS. Given the multifactorial nature of stuttering, we concur with Anderson and Ofoe (2019) that future research should also focus on the interaction between core EFs and domain-specific processes such as speech, language, motor, and emotion.

## Data availability statement

The raw data supporting the conclusions of this article will be made available by the authors, upon requesting them from the corresponding author.

## Ethics statement

The studies involving human participants were reviewed and approved by The National Bioethics committee of the Republic of Cyprus. Written informed consent to participate in this study was provided by the participants’ legal guardian/next of kin.

## Author contributions

All authors contributed to the design of the study. MP collected and analyzed the data as well as wrote the first draft of the manuscript. All authors contributed to the article and approved the submitted version.

## Funding

Data collection was funded by the Speech-Language Pathology Unit of the Psychology and Speech-Language Pathology Department of the University of Turku, Finland.

## Conflict of interest

The authors declare that the research was conducted in the absence of any commercial or financial relationships that could be construed as a potential conflict of interest.

## Publisher’s note

All claims expressed in this article are solely those of the authors and do not necessarily represent those of their affiliated organizations, or those of the publisher, the editors and the reviewers. Any product that may be evaluated in this article, or claim that may be made by its manufacturer, is not guaranteed or endorsed by the publisher.
